# Marginal Bone Maintenance and Different Prosthetic Emergence Angles: A 3-Year Retrospective Study

**DOI:** 10.3390/jcm11072014

**Published:** 2022-04-04

**Authors:** Diego Lops, Eugenio Romeo, Michele Stocchero, Antonino Palazzolo, Barbara Manfredi, Luca Sbricoli

**Affiliations:** 1 Department of Prosthodontics, Dental Clinic, School of Dentistry, University of Milan, 20142 Milan, Italy; diego.lops@unimi.it (D.L.); eugenio.romeo@unimi.it (E.R.); antonino.palazzolo@unimi.it (A.P.); barbara.manfredi@unimi.it (B.M.); 2 Department of Neurosciences, School of Dentistry, University of Padova, 35128 Padova, Italy; stocchero.michele@gmail.com

**Keywords:** dental implant, bone level, prospective study, sub-crestal placement, emergence profile

## Abstract

The aim of the present retrospective study was to assess marginal bone changes around implants restored with different prosthetic emergence profile angles. Patients were treated with implants supporting fixed dentures and were followed for 3 years. Marginal bone levels (MBL) measured at the prosthesis installation (t0) and at the 3-year follow-up visit (t1) were considered. The MBL change from t0 to t1 was investigated. Two groups were considered: Group 1 for restorations with an angle between implant axis and prosthetic emergence profile >30°, and Group 2 for those with an angle ≤30°, respectively. Moreover, peri-implant soft tissue parameters, such as the modified bleeding index (MBI) and plaque index (PI) were assessed. Seventy-four patients were included in the analysis and a total of 312 implants were examined. The mean EA in groups 1 and 2 was 45 ± 4 and 22 ± 7 degrees, respectively. The mean marginal bone level change (MBL change) of 0.06 ± 0.09 mm and 0.06 ± 0.10 mm were, respectively, in groups 1 and 2. The difference in the MBL change between the two groups was not statistically significant (*p* = 0.969). The MBL change does not seem to be influenced by the emergence angle for implants with a stable internal conical connection and platform-switching of the abutment diameter.

## 1. Introduction

Dental implants osseointegration is actually a well-established issue, due to improved surface characteristics. One of the modern implant therapy goals is to optimize esthetics, with a natural implant-supported restoration integration, especially regarding the peri-implant soft tissues. The correct interproximal papilla shape, the scalloping of the gingival margin and the thickness of the vestibular soft tissue are recognized as fundamental for esthetics. Even more important is the achievement of adequate cleansing procedures, in order to prevent any peri-implant soft tissues inflammation that may lead to a marginal bone level change. In fact, in the seventh *European Workshop on Periodontology*, different clinical parameters were recommended as items to evaluate the health status of the peri-implant tissue [1]: plaque accumulation, presence of bleeding on probing, probing depth and bone resorption, respectively.

The discrepancy between the implant diameter and the shape of the final prosthetic restoration requires compensation. According to *The Glossary of the Prosthodontic Terms*, 9th edition [2], the emergence angle (EA) is the angle between the average tangent of the transitional contour relative to the long axis of a tooth, dental implant or dental implant abutment.

Many studies have shown that over-contoured restorations may have an influence on gingival inflammation and plaque retention [3]. Excessive crown contour acts as a bacterial plaque accumulation factor [4], especially in the gingival third; therefore, proper oral hygiene is hindered. On the other hand, under-contoured restorations can be cleaned more easily with adequate tooth brushing techniques. In 2018, Katafuchi et al. [3] showed that an emergence angle (EA) of more than 30° is a significant risk factor for peri-implantitis and convex profiles create an additional risk for bone level implants, but not for tissue-level implants. A correlation between the restorations of EA and peri-implantitis was found in this study. Moreover, the wider the EA angle was, the greater the risk for peri-implantitis was found. Unfortunately, no data on the features of the implant to abutment connection are reported when different EAs were compared. In fact, only internal conical connection implants have proven superior to other configurations in achieving a tight seal and eliminating the microgap at the implant to abutment junction, and improvements in crestal bone maintenance [5] have been shown.

Considering the hypothesis that emergence angles of more than 30° could be a negative parameter for mid to long-term peri-implant tissues health, the aim of the present retrospective study was to analyze the influence of marginal bone level stability on restorations with different emergence angles (EA), for implants with an internal conical connection between the fixture and abutment. Moreover, how an EA angle may affect restorations in anterior or posterior areas differently, was investigated.

## 2. Materials and Methods

### 2.1. Patient Selection

Patients restored with implant fixed rehabilitation were included in the study; all cases have been treated with the same implant system (Anyridge, MegaGen Implant Co., Gyeongbuk, South Korea) between 2014 and 2017; moreover, radiographic and clinical parameters taken at the time of the prosthetic delivery were assessed, so that comparisons between baseline and 3 years follow-up visit were provided.

Patients with systemic diseases, a history of radiation therapy in the head and neck region, current treatment with steroids, a neurological or psychiatric handicap that could interfere with good oral hygiene, an immuno-compromised status (including infection with human immunodeficiency virus), severe clenching or bruxism, smokers (more than 15 cigarettes per day), drug or alcohol abuse and inadequate compliance were excluded.

All included patients gave their written consent after being informed in detail about the objectives of the study. Patients with single or multiple gaps requiring fixed implant-supported restoration were included. No exclusion criteria on the edentulous site were applied. Single and multiple restorations were included.

Along with the radiograph at the time of prosthetic delivery, the following data were collected: implant diameter and length, prosthesis type, implant site position, date of prosthetic delivery. Surgical and prosthetic procedures were performed by a single operator (DL) as below described.

The STROBE (Strengthening the reporting of observational studies in epidemiology; strobe-statement.org) guidelines checklist of items was followed.

### 2.2. Surgical Procedures

Before treatment, patients were clinically and radiographically evaluated. Panoramic and periapical radiographs were used as a first-level exam to evaluate the bone quantity before implant placement. If a second level exam was needed to assess the alveolar ridge width, due to a suspect bone deficiency, cone beam TC was performed. For each implant, a two-stage surgical technique was chosen. Implants were placed 1 to 2 mm below the crestal level [6], as recommended by the manufacturer, according to the scalloping of the bone crest.

An inter-implant distance of 3 mm at least, and/or an interproximal space from 1.5 to 3 mm between an implant and the adjacent tooth, were observed [7,8,9,10,11]. A periodontal probe was used to assess the correct distances.

Flaps were sutured over the implants to allow submerged healing. If slight horizontal dehiscence was present after an implant placement, a correction by means of xenograft bone granules (Geistlich Bio-oss, Wholusen, Switzerland) was performed. A resorbable collagen membrane (Geistlich Bio-gide, Wholusen, Switzerland) was used to stabilize the graft. If requested by patients, removable prostheses or provisional fixed bridges were temporarily used during the healing period to compensate for the edentulous gaps. Surgical re-entry was performed three months later, and transmucosal healing abutments were installed.

### 2.3. Prosthetic Protocol

Two weeks after surgical re-entry, an implant level impression was taken for screw-retained temporary restorations. Prostheses were inserted from one to six weeks after the implant level impression. After a period of 8 to 12 weeks for peri-implant soft tissue conditioning, a definitive implant level impression was taken.

Different types of fixed restoration were selected to restore patients’ edentulism: fixed single crown (SC), a partial fixed prosthesis (FPD) and full fixed prosthesis (FFD), respectively. Implants were located in the anterior jaw (central incisor to the first premolar) and in the posterior jaw (second premolar to the second molar). For cemented restorations, abutments were torqued down to 25 N/cm and restorations were cemented with temporary cement (Temp-Bond Clear, Kerr Corporation, Orange, CA, USA). On the other hand, for screw-retained prostheses, a torque of 25 N/cm was used to install the restorations by means of a proper torque wrench. After 2 weeks of loading, patients were recalled and an intraoral periapical radiograph of the restored implant site was taken; also, peri-implant clinical parameters were assessed.

### 2.4. Radiographic and Clinical Evaluations

All radiographs were taken with a standardized parallel technique with an X-ray apparatus supplied with a long cone and a Rinn Universal Collimator (Dentsply RINN, York, PA, USA) The following exposure parameters were used: 65–90 kV, 7.5–10 mA and 0.22–0.25 s. All radiographs were stored on a PC. Radiographic images were then analyzed with a software program (Image J, National Institute of Health, Bethesda, Rockville, MA, USA). Before measurement, each radiograph was calibrated by using the implant diameter and length as reference measures to correct any distortion.

Radiographic images were then analyzed with a software program (Image J, NIH, Montgomery County, MD, USA) to measure the following parameter: peri-implant bone level (marginal bone level, MBL). Measurements were made at the mesial and distal aspects of each implant and were reported in millimeters.

Because implants were sub-crestally positioned, measurements, where the bone crest was located coronally to the implant neck, were classified as negative values. On the contrary, measurements, where the bone crest was located apically to the implant neck, were classified as positive values.

For each implant-supported prosthesis, radiographs performed at the time of prosthetic delivery and at the follow-up visit were analyzed and compared to detect any change in the peri-implant marginal bone level. Such a procedure was carried out for each intraoral periapical radiograph by analyzing some reference measurements as: (i) implant neck diameter; (ii) implant length, by considering the distance between the implant neck and the most apical point of each implant, along an ideal line running parallel to the implant axis.

In addition, radiographs were used to measure the emergence angle (EA) between the implant long axis and the line tangent to the restoration, as described by Yotnuengnit et al. [12].

**Figure 1:**
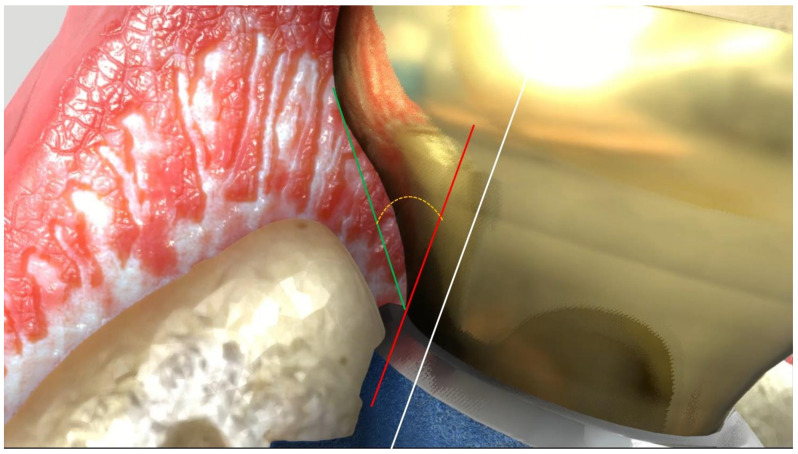
Emergence angle (EA) definition. White line: parallel to implant long axis. Red line: parallel to the white and tangential to the restoration from the implant to abutment connection. Green line: from implant to abutment connection point to the emergence profile. The angle of the intersection (yellow line) resulted in the emergence angle (EA).

**Figure 2:**
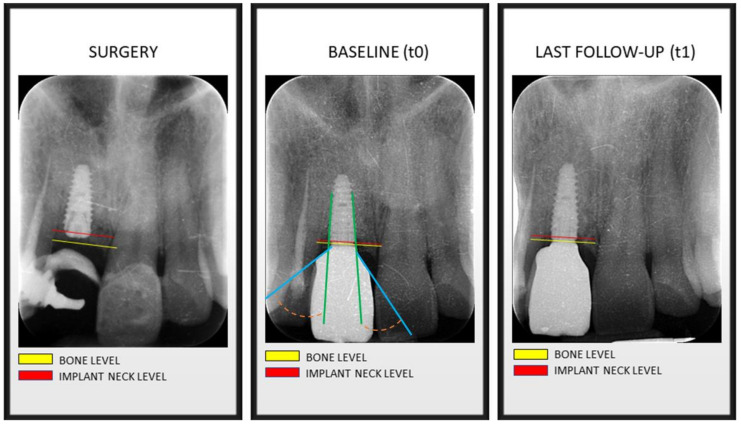
Bone levels at time of implant surgery, at prosthesis installation (baseline) and at last follow-up visit. Green line: implant axis. Blue line: prosthetic emergence profile axis. Orange line: angle between implant axis and prosthetic emergence profile >30°, determining the allocation in Group 1.

**Figure 3:**
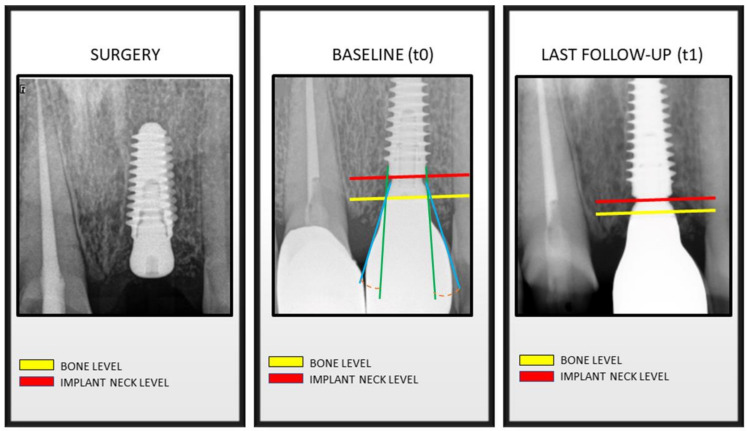
Bone levels at time of implant surgery, at prosthesis installation (baseline) and at last follow-up visit. Green line: implant axis. Blue line: prosthetic emergence profile axis. Orange line: angle between implant axis and prosthetic emergence profile ≤30°, determining the allocation in Group 2.

A line parallel to the implant’s long axis was drawn at the outer implant neck (Figure 1). A second one was drawn tangential to the restoration from the implant to abutment connection. The angle of the intersection resulted in the emergence angle (EA). Measurements were repeated twice. Group allocation was provided by considering the definitive restoration EA angle. EA > 30° were included in Group 1 (Figure 2); conversely, EA ≤ 30° were allocated to Group 2 (Figure 3). Since implants were placed sub-crestally, the transmucosal abutment was considered a part of the restoration.

A randomization was not performed because the choice of shape and emergence angle (EA) of each prosthesis was selected by the dental technician on the specific features of the edentulous site. For both MBL and EA parameters, mean values between the mesial and distal aspects were calculated to rate the respective measurements. A single operator (MS) performed all measurements. For the emergence angle measurement, intra-operator reliability was calculated.

Additionally, peri-implant soft tissue parameters, such as the modified sulcus bleeding index (mBI) and modified plaque index (mPI) [13,14] were assessed with a calibrated plastic probe (TPS probe, Vivadent, Schaan, Liechtenstein). Both mBI and mPI scores ranged from 0 to 3. Four sites for each implant (mesial, distal, buccal and lingual) were considered for recording probing depth scores. Moreover, for mBI and mPI indexes, mean values between the mesial and distal aspects were calculated to rate the respective measurements.

### 2.5. Statistical Analysis

Data were collected with an implant as a unit. Descriptive statistics were performed by calculating the mean and standard deviation for continuous variables and frequency distribution for categorical variables, respectively. The distribution of the outcome was assessed by the skewness values and by a normal quartile plot. Given the hierarchical structure of the data (i.e., implants nested within patients) a preliminary linear mixed model analysis (LMM) was conducted, to estimate the between-patients variation in the outcome variable (MBL change). Therefore, a random intercepts empty model was run: only the outcome variable (i.e., MBL change) was included and the intercept (i.e., MBL change mean) was allowed. No significant variation in random intercepts, var(u0) = 0.00, χ^2^(1) = 0.98, *p* = 0.33 was obtained. This result showed that the outcome variable did not vary across patients and confirmed the absence of cluster effects due to the hierarchical structure of the data. Thus, a linear regression approach with MBL as the dependent variable was calculated and adopted to evaluate the role of the type of prosthesis (SC, FPD, FFD), EA (Group 1 and Group 2) and implant site (anterior vs. posterior areas).

The descriptive statistics and the model processing were developed by a statistical software package (IBM SPSS Statistics for Mac, v. 22).

## 3. Results

Eighty patients (38 males and 42 females, respectively) aged from 22 to 84 years (mean age 55.6 ± 32.4 years) were recruited in the present study. During the follow-up period, 6 patients (3 males and 3 females, respectively) treated with 21 implants, on the whole, did not attend the 3 years follow-up visit, so these were considered drop-outs. Only 74 patients, consecutively followed in a 3 year period from the definitive prosthesis installation, were included in the present study.

A total of 312 implants were considered and the average follow-up period was 3.8 ± 1.3 years. Implants’ features of different sizes are reported in Table 1. Fixture distribution in the anterior or posterior area based on the EA type is reported in Table 2. The frequency of prosthesis type was as follows: 34 SC: single crown; 65 FPD: fixed partial denture; 12 FFD: fixed full denture. Anterior sites were considered from the first premolar to the contralateral. Conversely, implants placed in the second premolar and molar areas were included in the posterior subgroup, respectively.

The mean restorations EA in groups 1 and 2 were 45 ± 4 and 22 ± 7 degrees, respectively. EA values in Group 1 ranged from 31 to 47 degrees.

Mean marginal bone level changes (MBL change) of 0.06 ± 0.09 mm and 0.06 ± 0.10 mm were found, respectively, in groups 1 and 2 (Table 3). The MBL change in the two groups was not statistically different (*p* = 0.969). Moreover, when the MBL change of groups 1 and 2 were compared by considering the implant site (Table 3), no statistically significant difference was measured (*p* = 0.611 and 0.599, respectively, for anterior and posterior sub-groups).

Results from the linear regression for the MBL did not show a significant model using the selected parameters (type of prosthesis, EA and site location).

The mean MBI and PI values were recorded for both groups 1 and 2, respectively, at baseline and 3 years of follow-up control (Table 4).

The mean modified bleeding index changes (mBI change) were 0.2 and 0.1, respectively, in groups 1 and 2 (Table 3). Therefore, the mBI change in the two groups was not statistically different (*p* = 0.811). Similarly, modified plaque index changes (mPI change) were 0.2 and 0.2, respectively, in groups 1 and 2 (Table 5). Moreover, the mPI change in the two groups was not statistically different (*p* = 0.365).

## 4. Discussion

In the present retrospective study, the influence of the emergence angle (EA) on the marginal bone level was assessed for 312 implants placed in 74 patients after at least 3 years of function. The aim was to identify if a >30° EA might influence interproximal bone loss. The present findings partially agree with other recently published papers; a multivariate analysis to investigate the influence of prosthetic factors on the marginal bone level was conducted by Inoue et al. [15]. It was stated that there is no statistically significant correlation between the emergence angle and marginal bone level. In particular, the authors found that the marginal bone loss, after at least one year from prosthetic loading, was less for prostheses with an emergence angle between 20° and 40°. Such an outcome did not meet the present study findings, since the authors found bone stability with a mean value of 45° EA. The influence of the cervical coronal contour on marginal bone loss on 67 platform-switched implants was analyzed by Hentenaar et al. [16]. No statistically significant differences were reported between prosthetic emergence angles and marginal bone loss after 5 years of prosthetic loading. It must be recognized that only crowns with an emergence angle that did not exceed 18.7°, both on the mesial aspect and on the distal aspect, were analyzed. It is interesting, moreover, the analysis of platform-switched implants related to periodontal health parameters. The authors, in fact, found that after 5 years of prosthetic loading, the periodontal health parameters were very high, without any case of peri-implantitis. Additionally, no significant difference in both modified bleeding and plaque indices was measured at the last follow-up visit for groups 1 and 2 in the present study, respectively. Such clinical findings may show that adequate prosthetic emergence angles do not represent a risk factor for correct peri-implant soft tissues health, even if they are more than 30°.

Different results were found by Katafuchi et al. [3]. An emergence angle greater than 30° was judged to be correlated with an increased risk of peri-implantitis. However, the study by Katafuchi et al. [3] was conducted on bone-level non-platform-switched implants. Prosthetic rehabilitation on non-platform-switched bone-level implants may lead to excessively convex profiles where home hygiene maintenance is more difficult. Results comparable to those of Katafuchi were also found by Yi et al. [17]; they conducted a cross-sectional study on 349 implants 5 years after the prosthetic load in order to investigate the association between prosthetic factors and peri-implantitis. It was demonstrated that the emergence angle and emergence profile significantly affect the marginal bone level and the prevalence of peri-implantitis on bone-level implants, but not on tissue-level implants. Interestingly, the shape of the emergence profile on tissue-level implants was concave in the transmucosal part and convex in the part located above the mucosal margin.

In the present study, marginal bone loss was 0.06 mm for both EA groups after a minimum follow-up of 3 years. Such a finding agrees with previous studies [18,19] on bone stability around crestally and sub-crestally positioned implants with a platform-switching design.

The present study design suggests that the results should be interpreted with caution. One of the major drawbacks of such a clinical investigation was that the evaluation of the marginal bone level was made on the mesial and distal aspects of the implants, not taking into account the vestibular aspect. In fact, that kind of additional evaluation should require invasive 3D radiographs; for ethical reasons, such an approach was not possible to be achieved. In daily clinical practice, and particularly in the anterior area, it is now recognized that the vestibular-palatal position of the implant must be more palatal than the line that joins the center of the crowns of the adjacent teeth to allow an adequate thickness of vestibular bone [6]. This implant positioning may provide for an accentuated emergence angle if compared to the adjacent natural teeth. Another important limitation of the present study is the impossibility of controlling any confounders that could affect the stability of the marginal bone. A prospective analysis of such factors should be encouraged in the future.

## 5. Conclusions

Within the limits of the present investigation, with a tight and stable implant to abutment connection, an emergence angle of more than 30 degrees and less than 50 degrees may not influence the marginal bone levels’ stability. Nevertheless, despite the promising outcomes on the peri-implant hard tissues stability, more prospective and long-term data are required to confirm this trend.

## Figures and Tables

**Table 1:** Frequency of implant length and implant diameter.

		Diameter (mm)					Total
		3.5	4	4.5	5	6.5	
Length (mm)	7	2	2	7	6	11	28
	8.5	4	12	1	2	9	28
	10	10	35	34	11	1	91
	11.5	10	6	3	1	0	20
	13	16	51	53	4	0	124
	15	17	2	2	0	0	21
Total		59	108	100	24	21	312

**Table 2:** Frequency of implant distribution by site of placement.

			Position		Total
			Anterior	Posterior	
Jaw	maxilla	Count	92	89	181
		% of Total	29.5%	28.5%	58.0%
	mandible	Count	45	86	131
		% of Total	14.4%	27.6%	42.0%
Total		Count	137	175	312
		% of Total	43.9%	56.1%	100.0%

**Table 3:** MBL change for different groups (Group 1: EA > 30°; Group 2: EA ≤ 30°) by implant site (anterior and posterior). N: number of implants; SD: Standard Deviation.

	Group 1		Group 2	
	N	Mean ± SD	N	Mean ± SD
Anterior	95	0.066 ± 0.09 mm	42	0.057 ± 0.11 mm
Posterior	80	0.053 ± 0.10 mm	95	0.061 ± 0.10 mm
Total	175	0.060 ± 0.09 mm	137	0.060 ± 0.10 mm

Anterior: from first premolar to the contralateral one. Posterior: second premolar and molar area.

**Table 4:** MBI change for different groups (Group 1: EA > 30°; Group 2: EA ≤ 30°). N: number of implants; SD: Standard Deviation.

	Group 1			Group 2		
	Baseline Last Visit			Baseline Last Visit		
	N	Mean ± SD	Mean ± SD	N	Mean ± SD	Mean ± SD
Anterior	95	0.1 ± 0.3 mm	0.3 ± 0.2 mm	42	0.1 ± 0.2 mm	0.2 ± 0.2 mm
Posterior	80	0.3 ± 0.1 mm	0.5 ± 0.2 mm	95	0.2 ± 0.1 mm	0.4 ± 0.2 mm
Total	175	0.2 ± 0.2 mm	0.4 ± 0.3 mm	137	0.2 ± 0.2 mm	0.3 ± 0.3 mm

Anterior: from first premolar to the contralateral one. Posterior: second premolar and molar area.

**Table 5:** PI change for different groups (Group 1: EA > 30°; Group 2: EA ≤ 30°). N: number of implants; SD: Standard Deviation.

	Group 1			Group 2		
	Baseline Last Visit			Baseline Last Visit		
	N	Mean ± SD	Mean ± SD	N	Mean ± SD	Mean ± SD
Anterior	95	0.0 ± 0.0 mm	0.2 ± 0.3 mm	42	0.0 ± 0.0 mm	0.2 ± 0.2 mm
Posterior	80	0.1 ± 0.1 mm	0.3 ± 0.2 mm	95	0.3 ± 0.2 mm	0.5 ± 0.3 mm
Total	175	0.05 ± 0.1 mm	0.25 ± 0.3 mm	137	0.2 ± 0.09 mm	0.4 ± 0.3 mm
